# A New Species of *Hapalodectes* (Hapalodectidae, Mesonychia) from the Paleocene of Mongolia

**DOI:** 10.1134/S0012496623700709

**Published:** 2023-09-28

**Authors:** A. V. Lopatin

**Affiliations:** Borissiak Paleontological Institute, Russian Academy of Sciences, Moscow, Russia

**Keywords:** hapalodectids, mesonychians, Late Paleocene, Mongolia, lower molars, pulp recession, *Hapalodectes* evolution, species co-occurrence

## Abstract

*Hapalodectes paradux* sp. nov. (Hapalodectidae, Mesonychia) is described on the base of the dentary fragment with M_2_–M_3_ from the Tsagan-Khushu locality in Mongolia (Upper Paleocene, Naran Bulak Formation, Zhigden Member). The M_2_ and M_3_ are approximately the same size, with a high protoconid, anteriorly displaced reduced metaconid, anterolingually directed protocristid, very deep posterior notch, narrow talonid, and distinct hypoconid, entoconid and hypoconulid. Based on dental characters, the new species is presumably related to the base of the lineage of *Hapalodectes* that dispersed to North America at the beginning of the Eocene. Tsagan-Khushu is the only known locality where two species of *Hapalodectes* co-occur (larger *H. dux* Lopatin, 2001 and smaller *H. paradux* sp. nov.).

The Upper Paleocene Zhigden Member of the Naran Bulak Formation of the Tsagan-Khushu locality in southern Mongolia contains a rich assemblage of mammalian fossils [1, 2]. The finds of some groups are rare. These groups include hapalodectids, small mesonychians with well-pronounced carnivorous dental adaptations. *Hapalodectes dux* Lopatin, 2001 described from Tsagan-Khushu is based only on the holotype lower jaw with both tooth rows [3, 4]. *H. dux* was described as the earliest member of the genus (the faunal assemblage from the Zhigden Member is dated to the Late Paleocene, Gashatan) [1, 2]; at present, two more Paleocene species of *Hapalodectes* are known from China: *H. lopatini* Solé et al., 2017 (Middle Paleocene, Nongshanian, *Bothriostylops* Interval Zone) and *H. paleocenus* Beard et al., 2010 (Late Paleocene, Gashatan) [5, 6]. *H. hetangensis* Ting et Li, 1987 (*Orientolophus* Interval Zone) and *H. huanghaiensis* Tong et Wang, 2006 (*Homogalax* Interval Zone) inhabited China in the Early Eocene (Bumbanian), and *H. serus* Matthew et Granger, 1925 found there in the Middle Eocene (Irdinmanhan) [7–11]. *H. anthracinus* Zhou et Gingerich, 1991 (Wa-1 Zone) and *H. leptognathus* (Osborn et Wortman, 1892) (= *H. compressus* Matthew, 1909; zones Wa-4–Wa-7) are described from the Lower Eocene (Wasatchian) of the United States of America [12–16].

The description of a new find of *Hapalodectes* from the Zhigden Member of the Naran Bulak Formation of the Tsagan-Khushu locality (collections of the South Gobi party of the Joint Soviet-Mongolian paleontological expedition led by V.Yu. Reshetov, 1987) is presented below. The specimen is assigned to a new species, which is characterized by small size and a combination of primitive and advanced morphological features of the lower molars.

The studied material is stored in the Borissiak Paleontological Institute of the Russian Academy of Sciences (PIN) in Moscow. The illustrations were prepared using the Nikon D800 digital photo camera with an AF-S Micro NIKKOR 60mm f/2.8G ED lens, and the Neoscan N80 micro-CT-scanner at PIN. The scan parameters for the specimen PIN, no. 3104/775 were set at 84 kV, 48 μA, 5.5 μm pixel size, 180° rotation with 0.2° rotation step, and 0.1 mm Cu filter. The scan parameters for the specimen PIN, no. 3104/371 were 101 kV, 159 μA, 20 μm pixel size, 180° rotation with 0.2° rotation step, and 0.5 mm Cu filter. Acquired X-ray images (2800 × 2400 pixels) were processed with Neoscan software; 3D models were visualized in CTvox (Bruker microCT).

Order Mesonychia Matthew, 1937

Family Hapalodectidae Szalay et Gould, 1966

**Genus** ***Hapalodectes*** **Matthew, 1909**

***Hapalodectes paradux*** **Lopatin, sp. nov.**

**Etymology.** From Ancient Greek para, beside, and the species name *Hapalodectes dux*.

**Holotype.** PIN, no. 3104/775, right dentary fragment with M_2_–M_3_; Mongolia, South Gobi, Nemegt Basin, Tsagan-Khushu; Upper Paleocene, Naran Bulak Formation, Zhigden Member.

**Description** (Figs. 1, 2, 3b, 4b). The sizes are small for the genus. The lower molars are sectorial, strongly compressed transversely. The M_2_ and M_3_ have approximately the same size and similar structure (Fig. 1). The paraconid is half as high as the protoconid. At the base of the paraconid, there are two distinct additional cusps (anterolabial and anterolingual), delimiting the anterior reentrant groove and, together with it, providing strong tooth interlocking to the talonid of the more anterior tooth. The protoconid–metaconid region is separated from the paraconid and talonid by deep notches; the wide and very deep posterior notch is especially strongly developed. The protoconid is large and high, strongly compressed transversely, characterized by peculiar lanceolate outlines of the labial side. The protoconid trenchant blades (the preprotocristid and postprotocristid) are strong and form almost right angle (about 95° on M_2_, 100° on M_3_) in lateral view. The metaconid is mostly fused with the protoconid, reduced but distinct. The posterolingual side of the protoconid–metaconid regon lacks a clear emargination, emphasizing the metaconid distally. The proto-cristid directed anterolingually. The talonid is relatively narrow, significantly less in width than the trigonid. The strong  shearing crest of the talonid is divided in the middle by the protruding hypoconid apex. The reduced hypoconulid is located markedly below the hypoconid at the posterior end of the shearing crest of the talonid. The rudimentary entoconid appears as a tiny basal cuspule in the posterolingual part of the crown; on M_2_, it is located closer to the base of the crown and slightly larger than on M_3_. In lateral view, the posterior margin of the talonid of the M_2_ is oblique posterodorsally while that of the M_3_ is vertical.

**Fig. 1.**
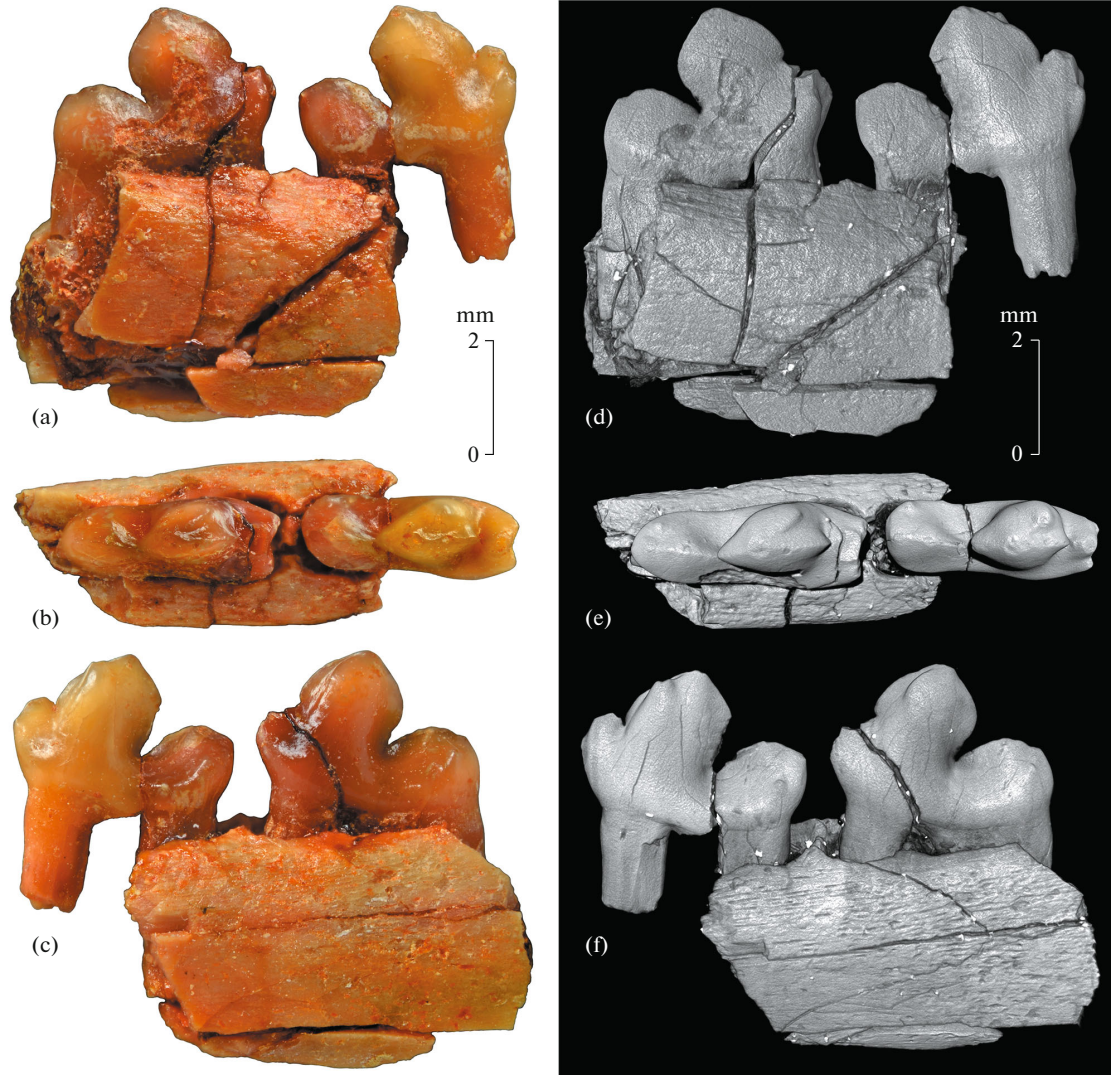
*Hapalodectes paradux* Lopatin, sp. nov., holotype PIN, no. 3104/775, right dentary fragment with M_2_–M_3_: (a–c) photographs; (d–f) CT model; (a, d) labial view; (b, e) occlusal view; (c, f) lingual view; Mongolia, Tsagan-Khushu; Upper Paleocene, Naran Bulak Formation, Zhigden Member.

**Fig. 2.**
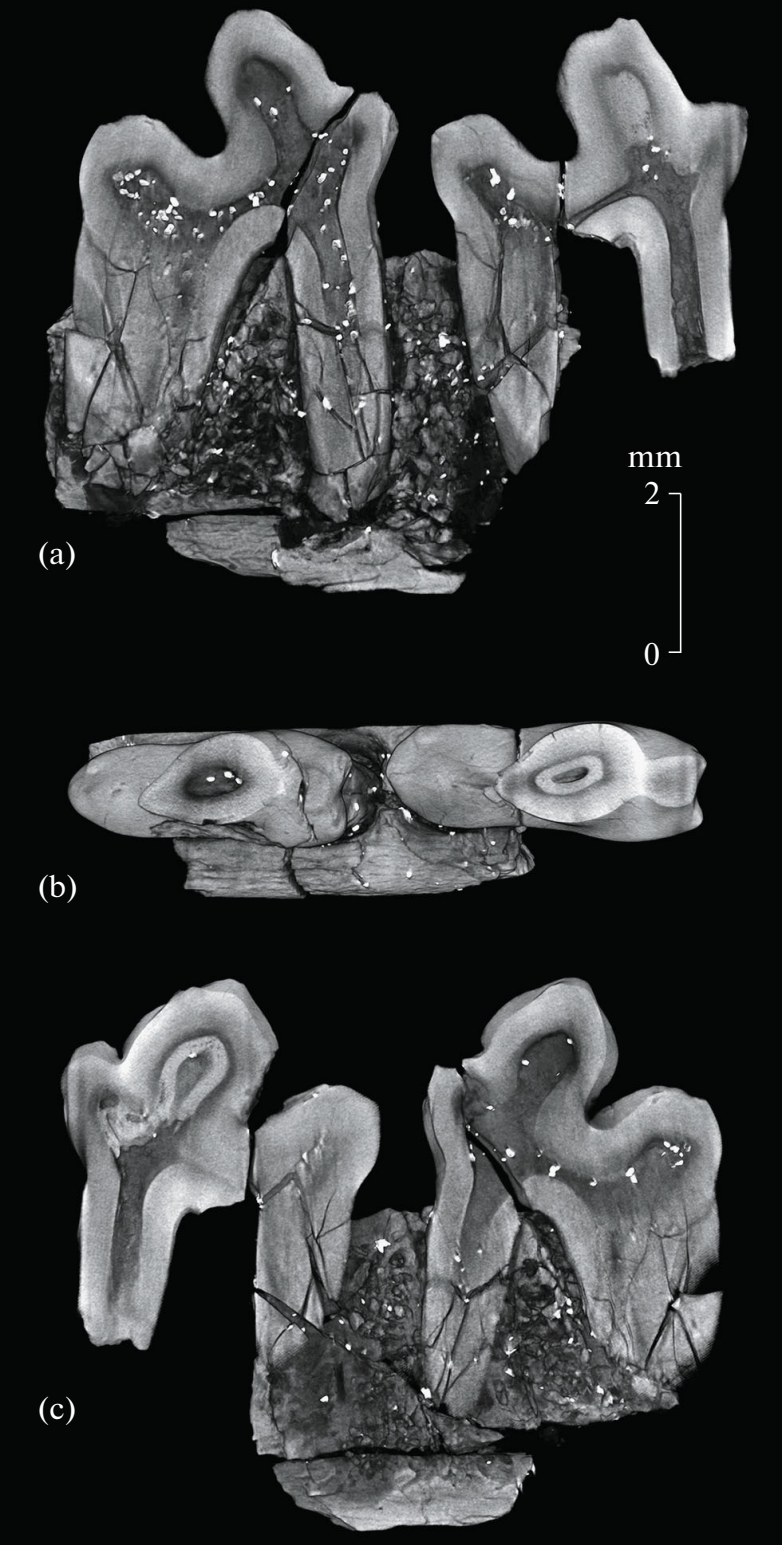
*Hapalodectes paradux* Lopatin, sp. nov., holotype PIN, no. 3104/775, right dentary fragment with M_2_–M_3_, CT model: (a) sagittal section, labial view; (b) frontal section ventral to the paraconid apices, occlusal view; (c) sagittal section, lingual view; Mongolia, Tsagan-Khushu; Upper Paleocene, Naran Bulak Formation, Zhigden Member.

**Fig. 3.**
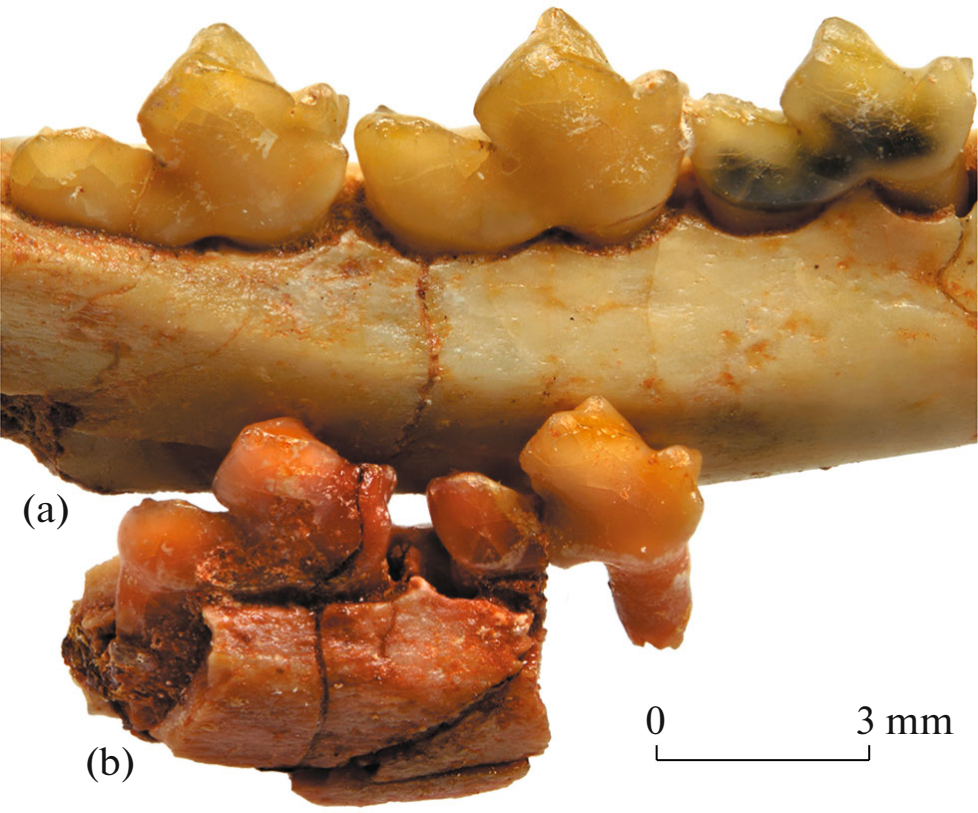
Comparison of two species of *Hapalodectes* from the Tsagan-Khushu locality in Mongolia: (a) *H. dux* Lopatin, 2001, holotype PIN, no. 3104/371, lower jaw, right M_1_–M_3_ region, dorsolabial view; (b) *H. paradux* Lopatin, sp. nov., holotype PIN, no. 3104/775, right dentary fragment with M_2_–M_3_, dorsolabial view; Upper Paleocene, Naran Bulak Formation, Zhigden Member.

**Fig. 4.**
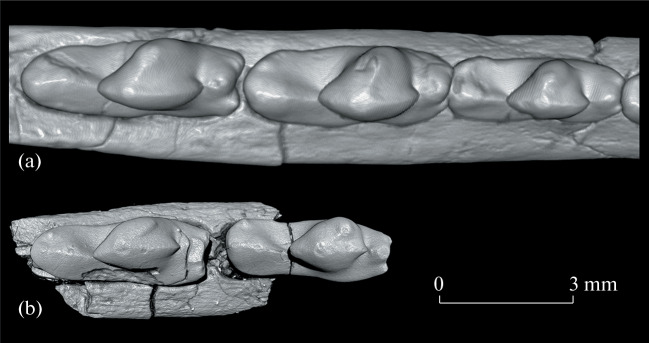
Comparison of the lower molar structure of two species of *Hapalodectes* from the Tsagan-Khushu locality in Mongolia, CT models, occlusal view: (a) *H. dux* Lopatin, 2001, holotype PIN, no. 3104/371, lower jaw, right M_1_–M_3_ region; (b) *H. paradux* Lopatin, sp. nov., holotype PIN, no. 3104/775, right dentary fragment with M_2_–M_3_; Upper Paleocene, Naran Bulak Formation, Zhigden Member.

On M_2_, the reduced metaconid has a distinct separate apex, which is strongly displaced anteriorly in relation to the protoconid apex and therefore clearly visible even from the labial side (Figs. 1a, 1d, 3b). The apices of the protoconid and metaconid are connected by the distinct elongated protocristid, which is strongly oblique anteriorly. In the horizontal plane, the angle formed by the protocristid and preprotocristid (the protocristid angle) is about 40° (Figs. 1e, 4b). The paraconid is equal in massiveness to the metaconid. The talonid widens distally and has a straight posterior margin.

On M_3_, the rudimentary metaconid is apically ridge-shaped, without a distinct separate apex, but the lingual metaconid swelling and protocristid are well developed. The protocristid angle is about 30°. The M_3_ noticeably exceeds the M_2_ in width of paraconid and region of additional cusps. The talonid tapers substantially backward and becomes rounded distally; it is longitudinally elongated more than on M_2_. The posterior root of the M_3_ is strongly expanded longitudinally at the base.

The internal structure of the teeth is characterized by a significant thickening of the dentin in the apical part of the protoconid–metaconid region, at the bottom of the posterior notch, and in the hypoconid area (Fig. 2a). On M_2_, the strong reduction of the pulp cavity (the pulp recession) in the trigonid region is fixed due to a visually distinct inner dentin layer (Figs. 2b, 2c); it may be associated with abnormally enhanced formation of the secondary dentin.

**Measurements** of the holotype, in mm. M_2_: length, 3.85; trigonid width, 1.45; talonid width, 1.35; protoconid height, 2.7; M_3_: length, 3.95; trigonid width, 1.5; talonid width, 1.3; protoconid height, 2.8.

**Comparison.** *Hapalodectes paradux* sp. nov. is markedly larger than *H. hetangensis*, but significantly smaller than all other described species of the genus (Table 1).

**Table 1.** Comparisons of the length of M_2_ and M_3_ (average values in parentheses, in mm) in *Hapalodectes* (the data on previously described species are given after [3, 5, 6, 9, 11, 14–16])

Species	M_2_	M_3_	M_3_/M_2_, %
*H. lopatini*	4.89	>5.0	>102
*H. paleocenus*	4.2	4.7	111.9
*H. dux*	4.5	4.4–4.5 (4.45)	98–100 (99)
*H. paradux* sp. nov.	3.85	3.95	102.6
*H. hetangensis*	3.1–3.4 (3.25)	3.1	91–100 (95.4)
*H. huanghaiensis*	4.9	5.0	102
*H. serus*	5.51	–	–
*H. anthracinus*	4.7–4.88 (4.79)	–	>100
*H. leptognathus*	5.4–6.01 (5.71)	5.9–6.37 (6.07)	106–110 (106.3)

The new species differs from the contemporaneous *H. dux* in smaller size (Fig. 3) and a number of features of the M_2_–M_3_ (Fig. 4), including a reduced metaconid strongly shifted forward (with a ridge-like apex on M_3_), a long protocristid, and a smaller protocristid angle (40° on M_2_, 30° on M_3_; in *H. dux* this angle is approximately 75° on M_1_, 65° on M_2_, and 60° on M_3_), as well as a deeper posterior notch and narrow talonid (in *H. dux*, the talonid is as wide as the trigonid).

*H. paradux* sp. nov. differs from *H. lopatini*, *H. paleocenus*, *H. anthracinus*, *H. leptognathus*, and *H. hetangensis* in approximately equal lengths of M_2_ and M_3_ (in the first four species, M_3_ is greatly increased, while in the latter species, it is slightly reduced).

The presence of the metaconid on molars distinguishes the new species from *H. anthracinus* and *H. serus*, the longer protocristid on M_2_–M_3_ and the ridge-like shape of the metaconid apex on M_3_ differs it from *H. paleocenus* and *H. leptognathus*, the presence of the entoconid distinguishes it from *H. huanghaiensis* and *H. serus*, and the relatively slight reduction of the latter cusp differs it from *H. hetangensis*, *H. anthracinus*, and *H. leptognathus*.

*H. paradux* sp. nov. differs from species that retain a relatively well-developed metaconid on molars (see Table 2) by its greater reduction, strong anterior displacement and, accordingly, by the sharper protocristid angle, as well as the ridge-shaped apex of this cusp on M_3_.

**Table 2.** Comparisons of the structure of M_2_ and M_3_ in *Hapalodectes* (data on previously described species are given after [3, 5, 6, 9, 11, 14–16]; protocristid angle measured from images)

Species	Metaconid	Protocristid angle		Entoconid
		M_2_	M_3_	
*H. lopatini*	Developed	55°	–	– (developed on M_1_)
*H. paleocenus*	Reduced	50°	45°	Rudimentary
*H. dux*	Developed	65°	60°	Rudimentary
*H. paradux* sp. nov.	Reduced	40°	30°	Rudimentary
*H. hetangensis*	Developed	–	–	Rudimentary
*H. huanghaiensis*	Developed	60°	–	Absent
*H. serus*	Absent	Absent		Absent
*H. anthracinus*	Absent	Absent		Rudimentary
*H. leptognathus*	Reduced	–	–	Rudimentary or absent

**Remarks.** Findings of hapalodectids are quite rare, and their diversity in each locality is usually limited to one species. Currently, Tsagan-Khushu is the only locality where two species of *Hapalodectes* co-occur (larger *H. dux* and smaller *H. paradux* sp. nov.).

The holotypes of *H. dux* and *H. paradux* sp. nov. differ greatly in the size and dental structure and therefore cannot be sex- and age-related or other intraspecific variations. Sexual dimorphism in *Hapalodectes* is presumably reflected in the depth of the horizontal ramus of the mandible [6].

The calculation according to the formula proposed by Zhou (Y = 1.327 × X – 3.355, where X = ln(L × B) (L, length of M_2_; B, width of M_2_, mm) and Y = ln W (weight, kg)) [17] made it possible to estimate the body mass of *H. paradux* sp. nov. in 360 g. The body mass calculated using this formula is 700–1100 g (mean 900 g) for *H. leptognathus*, 870 g for *H. serus*, 740 g for *H. lopatini*, 670 g for *H. huanghaiensis*, 530 g for *H. anthracinus*, 500 g for *H. dux*, 460 g for *H. paleocenus*, and 190–200 g for *H. hetangensis* [3, 6, 17]. According to the size of the postcranial skeleton bones, the body mass of *H. leptognathus* is estimated at 1000–1500 g [18].

**Material.** Holotype.

The recent phylogenetic analysis of the mesonychians (Mesonychia, or Acreodi), archaic carnivorous ungulates from the Paleogene of the Northern Hemisphere, suggests the following divergence order of the clades of *Hapalodectes* ([6], fig. 3, majority-rule consensus tree): (*H. lopatini* + *H. dux*) ((*H. hetangensis* + *H. huanghaiensis*) (*H. paleocenus* (*H. leptognathus* (*H. anthracinus* + *H. serus*)))). This evolutionary scenario implies the basal position of the Middle Paleocene *H. lopatini* and the Late Paleocene *H. dux*, based on a large number of primitive features of these early Asian species. Their lower molars are characterized by a well-defined metaconid, relatively large additional cusps in front of the paraconid, a wide talonid, and distinct talonid cusps (the hypoconid, hypoconulid, and entoconid). The ultimate lower molar M_3_ is approximately equal in length to the M_2_ in *H. dux* and slightly longer than that in *H. lopatini*.

Evolutionary more advanced species are divided into two main clades [3, 5, 6]. The first of them includes the Early Eocene *H. hetangensis* and *H. huanghaiensis* from China, the second comprises the Late Paleocene *H. paleocenus* from China, the Early Eocene *H. anthracinus* and *H. leptognathus* from North America, and the Middle Eocene *H. serus* from China.

The close relationship between *H. hetangensis* and *H. huanghaiensis* is supported by the similar structure of the upper molars, as well as the presence of a well-defined metaconid apex and relatively large additional anterior cusps on the lower molars [6]. The entoconid is rudimentary in *H. hetangensis* and absent in *H. huanghaiensis*; the hypoconid and hypoconulid are distinct. The M_3_ is equal in length to the M_2_ in *H. huanghaiensis* and inferior to the latter in *H. hetangensis*.

The second clade is characterized by relatively large size, small crestiform additional anterior cusps, and progressive reduction of the metaconid and entoconid up to their complete disappearance in *H. serus* (see Table 2). In members of this lineage, the M_3_ is significantly longer than the M_2_ (this is not known for *H. serus*), and talonids are relatively narrow, as in *H. paradux* sp. nov. The distinct reduction of the metaconid (especially on M_3_), recorded in *H. paradux* sp. nov., suggests that the new species is close to this *Hapalodectes* lineage. This reduction was associated with a decrease in the width of the middle part of the lower molars (to enhance their shearing action) and occurred through the fusion of the metaconid base with the protoconid. The narrowing of the protoconid–metaconid region in *H. paradux* sp. nov. included the anterior displacement of the metaconid apex, decreasing the protocristid angle (also occurred in *H. leptognathus*, see [13, 14]). However, the new species has retained some features that are primitive for the genus, namely, relatively large additional anterior cusps, presence of the entoconid and approximately the same length of the ultimate and penultimate lower molars. Based on the combination of the dental features, *H. paradux* sp. nov. can be considered as a sister species to *H. paleocenus*.

Thus, four species of *Hapalodectes* are now known from the Paleocene of Asia (*H. lopatini*, *H. dux*, *H. paleocenus*, and *H. paradux* sp. nov.) that confirms the conclusion about a fairly bushy Paleocene radiation of this group [6]. The scenario of the evolution of the genus in the Eocene includes the following assumptions. The *H. hetangensis* and *H. huanghaiensis* lineage, endemic to southeastern China, may be related in origin to this region, from which the Middle Paleocene *H. lopatini* is also known [6]. At the beginning of the Eocene, members of another lineage dispersed to North America, producing local species radiation on the new continent (*H. anthracinus* and *H. leptognathus*). The relatively small-sized Late Paleocene *H. paleocenus* and *H. paradux* sp. nov., inhabited the area of the Mongolian Plateau, could be at the base of this lineage. The common ancestor of the latter two species, in its turn, apparently appeared as a result of the earlier Paleocene radiation, which also gave rise to *H. dux*. At an earlier stage of evolution, there was the split of the ancestor of all the discussed species and *H. lopatini*.

The special role of the Mongolian Plateau area in the Late Paleocene radiation of *Hapalodectes* [3, 6] should be noted in connection with the existence of three species of this genus here in the Gashatan, namely, *H. paleocenus*, *H. dux*, and *H. paradux* sp. nov., and the co-occurrence of the latter two in the Tsagan-Khushu locality. Apparently, the significant dissimilarities in the structure of molars and body size in coexisting species *H. dux* and *H. paradux* sp. nov. contributed to the minimization of trophic competition between them. This corresponds to the concept of morphological divergence in dentition of carnivorous mammals as a factor in sympatric speciation [19] and emphasizes the role of the Mongolian Plateau area as the probable center of the Late Paleocene diversification of hapalodectids.

It is assumed that at the beginning of the Middle Eocene, a member of the North American lineage of *Hapalodectes* migrated back to Asia, where it gave rise to *H. serus* [3, 5, 6]. However, it cannot be ruled out that *H. serus* arose as a result of the parallel development of an Asian lineage of *Hapalodectes*, which in the Middle Eocene independently reached the same level of specialization as the North American *H. anthracinus* did in the Early Eocene [3]. In this part, the evolutionary scenario can be significantly corrected and detailed when the remains of evolutionary more advanced members of the *H. hetangensis* and *H. huanghaiensis* lineage or other descendants of the Paleocene radiation of *Hapalodectes* are found in the Lower and Middle Eocene of Asia.
