# Erratum to: **A New Species of** ***Hapalodectes*** **(Hapalodectidae, Mesonychia) from the Paleocene of Mongolia**

**DOI:** 10.1134/S0012496623050101

**Published:** 2024-01-25

**Authors:** A. V. Lopatin

**Affiliations:** Borissiak Paleontological Institute, Russian Academy of Sciences, Moscow, Russia

The article “A New Species of *Hapalodectes* (Hapalodectidae, Mesonychia) from the Paleocene of Mongolia,” written by A.V. Lopatin, was originally published Online First in Springer-Link on September 28, 2023 without Open Access. After publication, the author decided to make the article an Open Access publication. Therefore, the copyright of the article has been changed to © The Author(s) 2023 and the article is forthwith distributed under the terms of a Creative Commons Attribution 4.0 International License (http://creativecommons.org/licenses/by/4.0/, CC BY), which permits use, duplication, adaptation, distribution and reproduction of a work in any medium or format, as long as you cite the original author(s) and publication source, provide a link to the Creative Commons license, and indicate if changes were made.

